# Targeting Activated Pathways in Doxorubicin-Resistant TNBC Alters Signaling, Survival and EMT: A Double-Edged Sword

**DOI:** 10.3390/ijms27062792

**Published:** 2026-03-19

**Authors:** Irem Dogan Turacli, Sahika Cingir Koker, Kubra Paspal Eroglu, Banu Yalcin

**Affiliations:** 1 Faculty of Medicine, Department of Medical Biology, Ufuk University, Ankara 06520, Turkey; sahika.koker@gmail.com (S.C.K.); kubra.paspal.eroglu@gmail.com (K.P.E.); 2 Ogretmen Naime Tomek Research Laboratory (ONTAL), Faculty of Medicine, Ufuk University, Ankara 06520, Turkey; banuyalcnn@gmail.com

**Keywords:** TNBC, doxorubicin, MEK, PI3K, EMT

## Abstract

Triple-negative breast cancer (TNBC) poses significant therapeutic challenges due to the limited availability of targeted treatment options and the development of resistance to chemotherapy, including doxorubicin (DOX). The objective of this study was to investigate the impact of inhibiting activated pathways in DOX-resistant TNBC and examine the effects on MAPK and PI3K/Akt signaling pathways, cell cycle regulation, and the regulators of the epithelial–mesenchymal transition (EMT) process. Continuous exposure of cells to increasing concentrations of DOX resulted in the selection of resistant cells that exhibited EMT characteristics. We assessed the expression levels of markers related to cell death, survival, mitophagy pathways and EMT using Western blotting and qPCR in both sensitive and resistant cells with activated-pathway inhibitor treatments. Additionally, we demonstrated differences in migration capacity between resistant and sensitive cells with or without inhibitor treatments. It was found that MEK inhibition was less effective than PI3K inhibition in both sensitive and resistant cells. Expression analyses clearly demonstrated that resistant cells exhibited more aggressive behavior, as indicated by EMT- and survival-related gene expressions. The combination of MEK and PI3K inhibitors was more effective in shutting down these signals in both cell types. The ability to induce EMT in DOX-resistant cells revealed that one form of resistance might combine with another, acting as a mediator for cellular switch. Although drug resistance and various inhibitors reduce the proliferative capacity of cells and related parameters, resistance contributes to the acquisition of metastatic characteristics.

## 1. Introduction

Breast cancer (BC) is the most common type of cancer in women worldwide. It is genetically and clinically heterogeneous and primarily classified into two subgroups: ductal carcinoma in situ (DCIS) and lobular carcinoma in situ (LCIS). Histopathological type, tumor stage, and molecular markers such as Estrogen Receptor (ER), Progesterone Receptor (PR), and Human Epidermal Receptor 2 (HER2) are used for both the classification of breast cancer and the development of treatment strategies [1]. Triple-negative breast cancer (TNBC) is the most aggressive subtype of breast cancer, characterized by the lack of expression of ER, PR and HER-2 [2]. The aggressive nature of TNBC is attributed to its heterogeneity, its capacity for rapid metastasizing to the brain, lungs, and bones, and its rapid onset of recurrence. These characteristics challenge the treatment for TNBC [3]. Due to the absence of hormone receptor expressions in TNBC, it precludes the possibility of endocrine therapy, thus rendering chemotherapy and radiotherapy as the main therapeutic options for TNBC [4]. Lately, immune checkpoint inhibitors (ICI) have been investigated but the efficacy of ICI therapy has been found to be dissatisfying compared to the sustained therapeutic responses of other tumor types [5]. Therefore, it is important to determine how to improve the effectiveness of therapies or overcome their disadvantages for TNBC patients.

As a topoisomerase II inhibitor, doxorubicin (DOX) prevents DNA replication and triggers cell death, autophagy, or senescence by interfering with DNA repair and RNA synthesis through DNA intercalation, which results in DNA double-strand breaks [6]. It also contributes to reactive oxygen species (ROS) production. Excessive ROS levels seriously damage the mitochondrial structure, which eventually leads to apoptosis [6,7].

The efficacy of doxorubicin is diminished in cancer cells due to multiple mechanisms of resistance. Some remarkable mechanisms include expression levels of topoisomerase II [8] and overexpression of ATP-binding cassette transporters, such as P-glycoprotein, which pump the drug out of the cells [9]. Also, upgraded DNA repair mechanisms, especially homologous recombination repair or anti-apoptotic pathways, can play significant roles for resistance [10,11,12].

Ras/Raf/MEK/ERK and PI3K/AKT/mTOR are the two main survival signaling pathways that can also be activated to help resistant cells to avoid the effects of several agents. Due to its adaptive function in protecting cancer cells from oxidative stress, the MAPK/ERK pathway has been demonstrated to promote DOX resistance [13]. By phosphorylating several downstream substrates involved in gene transcription, protein synthesis, cell survival, cell cycle, and metabolism, the PI3K/Akt/mTOR pathway can induce drug resistance [14,15]. Also, gain-of-function mutations in the effectors of these mentioned pathways or loss-of-function mutations in their negative regulators can account for the resistance mechanisms.

DOX-mediated DNA damage can cause cell cycle arrest, leading to apoptosis [16]. However, the mutation status and expression levels of apoptosis regulators and DNA-damage-sensing tumor suppressor proteins such as p53 play a crucial role in cell cycle regulation and activation of intracellular signaling pathways [17,18,19]. Moreover, due to a feedback mechanism, these survival pathways may counteract the inhibition of cell cycle arrest and apoptosis even on high doses of DOX, thereby contributing to increased DOX resistance. Also, drug resistance has been linked to the epithelial–mesenchymal transition (EMT) process, which inhibits apoptosis and prevents senescence. The cytoskeletal protein Vimentin and the cell surface protein E-Cadherin are biomarkers of the EMT process [20]. The MAPK/ERK and PI3K/Akt signaling pathways mentioned above can also trigger EMT mechanism and contribute to drug resistance [21].

One of the mechanisms related to DOX resistance is mitophagy, which is an essential quality control mechanism that uses autophagy to specifically break down damaged or excessive mitochondria [22]. DOX causes oxidative stress and mitochondrial dysfunction in cancer cells. To survive, cells may activate mitophagy to clear the damaged mitochondria [23]. In the canonical mitophagy pathway, under mitochondrial stress, PINK1 accumulates on damaged mitochondria and recruits Parkin, an E3 ubiquitin ligase. Parkin ubiquitinates mitochondrial proteins, targeting them for autophagic degradation [24,25]. Also, there are alternative mitophagy mechanisms (e.g., BNIP3-, NIX-, or FUNDC1-mediated pathways). These non-canonical pathways can be hypoxia- or stress-responsive, which are conditions common in drug-resistant tumors [26,27].

Understanding how all these intracellular signaling pathways contribute to drug resistance will help therapeutic approaches work better and prevent treatment failure. Therefore, the purpose of this work was to clarify how DOX resistance affects apoptosis, the MAPK and PI3K/Akt signaling pathways, cell cycle control, the canonical mitophagy pathway and EMT process regulators in TNBC. Additionally, since the PI3K and MAPK pathways are upregulated in resistance, we explored how PI3K and MEK inhibitors affected these specified pathways. Firstly, we assessed cellular proliferation in the presence of inhibitors targeting the MAPK and PI3K/Akt signaling pathways in DOX-resistant murine TNBC cells. Additionally, we validated RNA and protein expression levels and analyzed cell migration under all aforementioned conditions. Our findings indicate that the adaptive function of the MAPK/ERK pathway may serve as a protective mechanism for DOX-resistant TNBC cells against cell death pathways while also contributing to their aggressive phenotype. Inhibition of these activated pathways may enhance the efficacy of DOX and mitigate the risk of treatment failure.

## 2. Results

**Figure 1:**
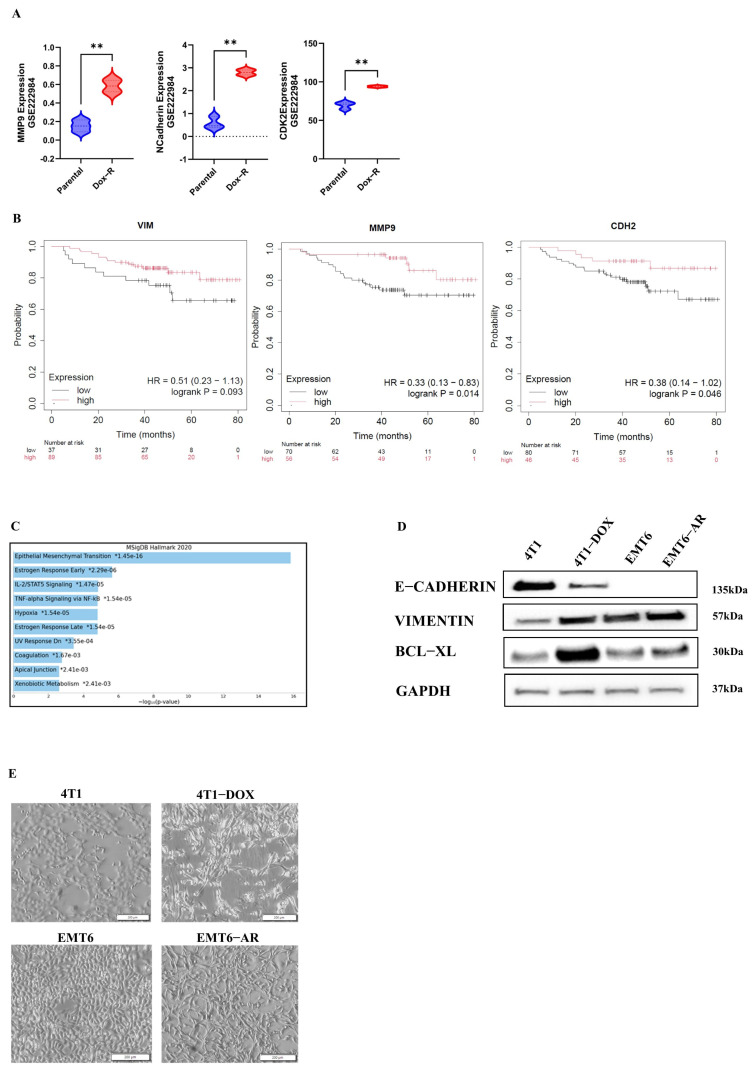
The relation between DOX resistance and metastatic characteristics in TNBC. (**A**) In DOX-resistant MDA-MB-231 TNBC cells, MMP9, N-Cadherin and CDK2 expressions were significantly upregulated in publicly available datasets, ** *p* < 0.01. (**B**) In TNBC patients, high Vimentin/MMP9/CDH2 (N-Cadherin) expressions were correlated with lower survival rates. (**C**) Analysis of the top 250 genes extracted from these datasets revealed that the EMT pathway is the most prominently impacted. (**D**) The increased expression of key EMT markers in DOX-resistant 4T1 and EMT6 cells. (**E**) Microscopic images of sensitive and DOX-resistant cells (10×).

Since doxorubicin resistance is associated with the EMT process, we first aimed to assess the expression levels of certain EMT markers in publicly available datasets. One such dataset, GSE222984, includes an analysis of both doxorubicin-resistant TNBC MDA-MB-231 cells and their parental counterparts. Our analysis revealed that the expression levels of MMP9 and N-Cadherin were significantly elevated in the DOX-resistant cells. In addition, we observed an increase in the expression of CDK2, a key regulator of the cell cycle, in the DOX-resistant cells as well (Figure 1A). Additionally, Kaplan–Meier analysis in TNBC patients revealed that high expressions of Vimentin, MMP9, and CDH2 (N-Cadherin) are associated with poorer survival rates (Figure 1B). When the top 250 genes were extracted from the GSE202536 dataset and analyzed in Enrichr, the EMT pathway emerged as the most significantly affected pathway (Figure 1C).

To elucidate the molecular and morphological effects of DOX resistance on cells, we conducted an analysis of the expression of key EMT markers, such as E-Cadherin and Vimentin, using the Western blot technique in both parental and resistant cell lines (Figure 1D). Additionally, microscopy images were obtained (Figure 1E). As depicted in Figure 1D,E, a distinct EMT phenotype is observed in DOX-resistant TNBC murine cells.

**Figure 2:**
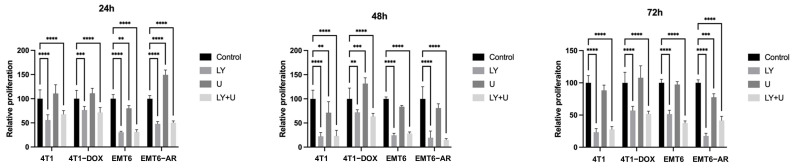
Proliferation analysis of DOX-sensitive and -resistant 4T1 and EMT6 cells treated with PI3K inhibitor LY294002 (10µM) and MEK inhibitor U0126 (10µM) for 24, 48, and 72 h. Statistical significance is indicated as follows: ** *p* < 0.01, *** *p* < 0.005, **** *p* < 0.001.

Since activation of AKT and MAPK pathways plays a crucial role in DNA-damaging drug resistance, we analyzed the inhibition of these pathways in DOX-resistant TNBC cells. Firstly, we performed a viability assay to assess the proliferative response of both parental and DOX-resistant cells to PI3K(LY294002) and MEK (U0126) inhibitors. As shown in Figure 2, the cells exhibited a higher sensitivity to the PI3K inhibitor, LY294002. Each group was normalized to its respective control group and statistical analysis was performed only within each group. However, based on the comparison of the parental and resistant group graphs, it is evident that for 4T1 cells, DOX resistance induces a more aggressive proliferative behavior, even in the presence of inhibitors, and the cells exhibit greater viability under these conditions at 24, 48 and 72 h. Additionally, the MEK inhibitor U0126 was generally ineffective in modulating proliferation in both parental and DOX-resistant cell lines. Although this effect diminished in the subsequent hours, at 24 h, it caused a significant increase in proliferation in EMT6-AR cells. When the two inhibitors combined, relative proliferation was similar to LY294002 alone in almost all groups, which indicates that MEK inhibition is not an effective strategy to reduce viability in TNBC cells.

**Figure 3:**
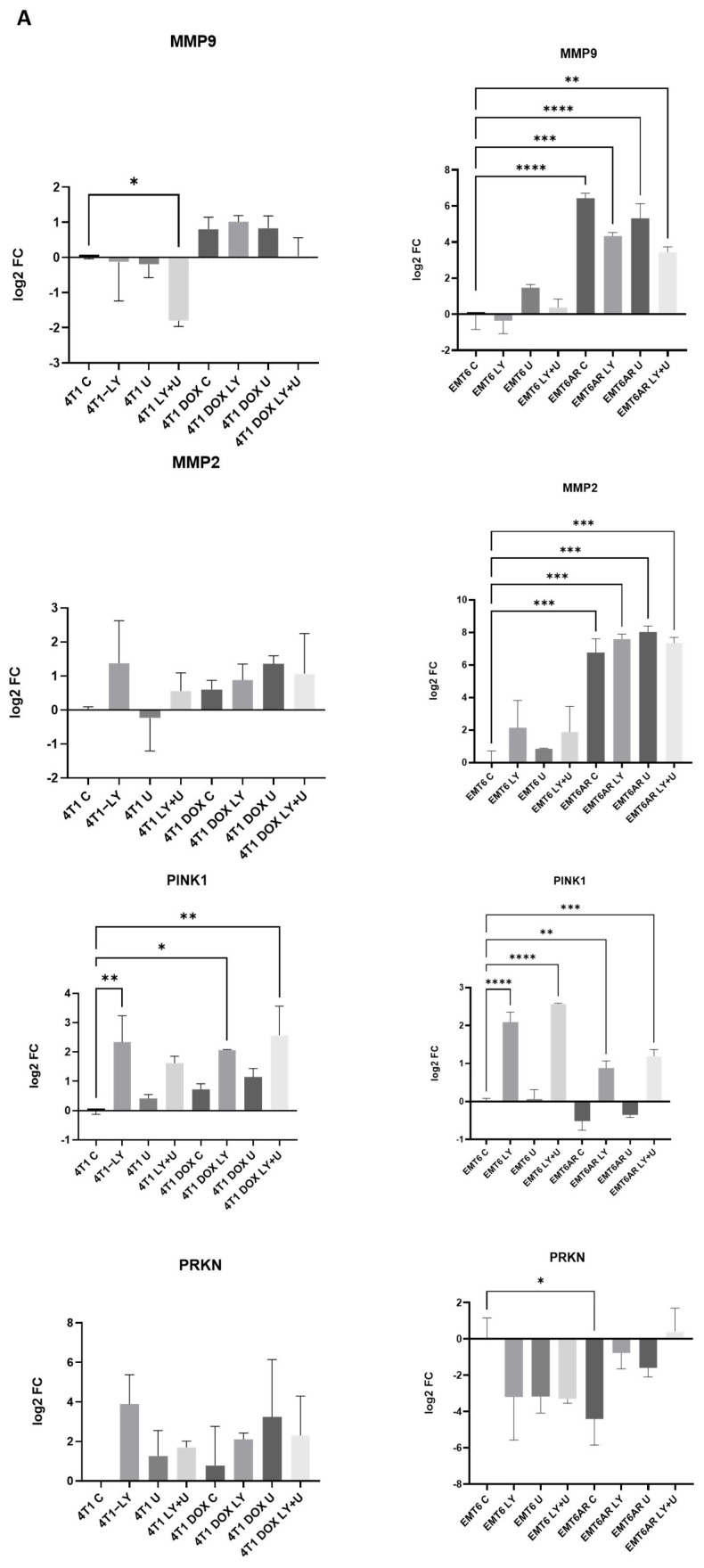

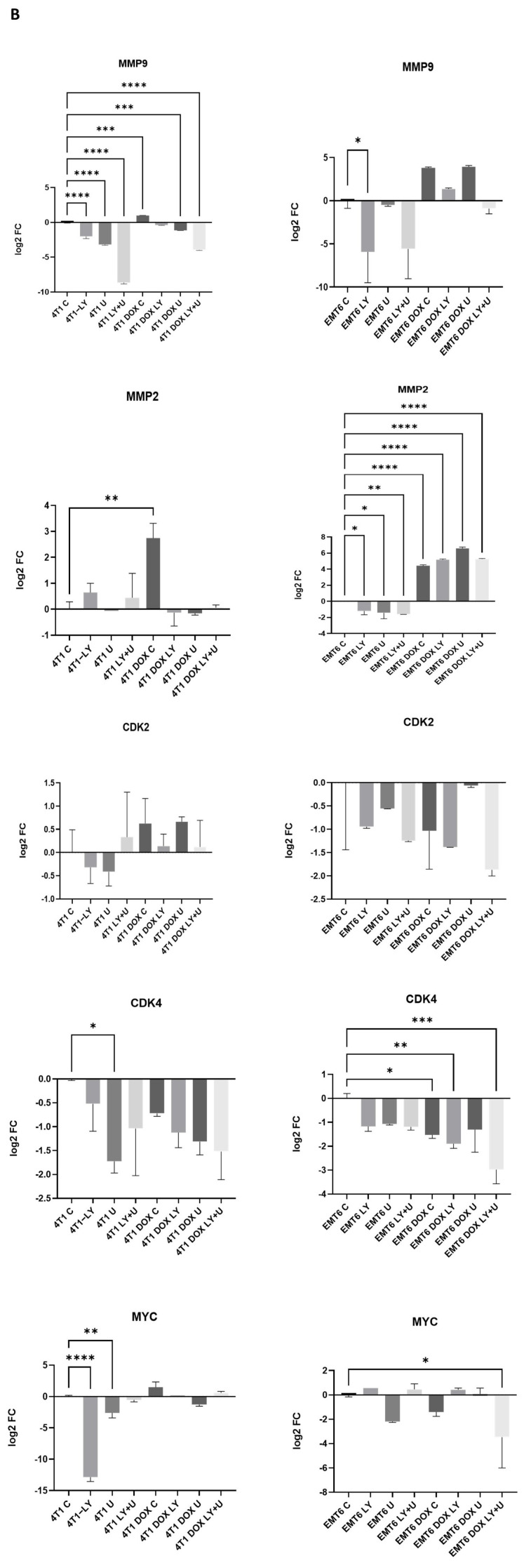
Relative expression levels of selected genes in DOX-sensitive and -resistant 4T1 and EMT6 cells after treatment with PI3K and MEK inhibitors for 24 h (**A**) and 48 h (**B**). Statistical significance is indicated as follows: * *p* < 0.05, ** *p* < 0.01, *** *p* < 0.005, **** *p* < 0.001.

To further investigate the proliferation-, mitophagy- and EMT-related changes in greater detail, we conducted qPCR analysis for well-established markers: CDK2, CDK4, MYC, PINK, PARKIN, MMP2 and MMP9. All groups were evaluated relative to their respective parental control groups. As shown in Figure 3A, after 24 h of inhibitor treatment of both parental and resistant cell lines, MMP9 gene expression was higher in all PI3K-inhibitor- and MEK-inhibitor-treated DOX-resistant 4T1 cells compared to their parental control. Also, combined treatment decreased MMP9 expression in parental 4T1 group. Furthermore, MMP2 expression was higher in the DOX-resistant group. In the case of EMT6 cells, MMP9 and MMP2 expressions were notably higher in all DOX-resistant groups with or without treatments, which shows a clear transition. Also, inhibitor treatments could not reduce the expression levels of MMP2 or MMP9 in EMT6AR cells significantly.

When we examined the expressions of the mitophagy markers PINK1 and PARKIN at 24 h in DOX-sensitive and-resistant cells with or without inhibitor treatment, we observed an increase in PINK1 expression upon LY294002 administration in both 4T1 and EMT6 cell lines. However, although this increase was statistically significant in all EMT6 groups compared to parental control, it was markedly attenuated in the DOX-resistant group. In 4T1 cells, PARKIN expression increased in both inhibitor-treated and DOX-resistant cells, but this increase was not statistically significant. In EMT6 cells, PARKIN expression was significantly reduced only in the resistant cells without inhibitor treatment, compared to the parental control (Figure 3A).

Regarding the expression changes of these EMT and mitophagy markers, we wanted to see how these markers and the cell cycle regulators change after 48 h exposure to the inhibitors. As seen in Figure 3B, MMP9 expression was downregulated with inhibitors and upregulated in DOX-resistant cells in 4T1 cells. However, combined treatment of PI3K and MEK inhibitors reduced its expression. On the other hand, although LY294002 significantly downregulated MMP9 expression in EMT6 at 48 h, its expression was not significantly affected in DOX-resistant cells. At 48 h in 4T1 cells, a significant increase in MMP2 expression was observed only in the DOX-resistant group, whereas no significant changes were detected in the other groups. In EMT6 cells, MMP2 expression decreased in the DOX-sensitive group upon inhibitor treatment, while it significantly increased in the DOX-resistant group, and this increase was not affected by the inhibitor treatments.

The expression of cell cycle regulator CDK2 did not show any significant differences between the groups in any of the cell lines. However, the expression of CDK4 significantly decreased in 4T1 cells upon MEK inhibitor treatment, whereas in EMT6 cells, a significant reduction was observed across nearly all groups within the DOX-resistant groups compared to the parental control (Figure 3B).

In 4T1 cells, MYC was significantly downregulated in the PI3K-inhibitor-treated group at 48 h. Although the expression of MYC was higher in the DOX-resistant control group compared to the parental group, there was no significant difference between the groups in terms of MYC expression in 4T1 cells. On the other hand, the expression of MYC exhibited fluctuations among the groups in EMT6 cells, which makes it difficult to identify a clear pattern. However, in DOX-resistant EMT6-AR cells treated with both LY294002 and U0126, a significant decrease in expression was observed. We hypothesize that the observed differences in proliferation and expression responses between the groups are likely due to genomic and cellular signaling alterations.

**Figure 4:**
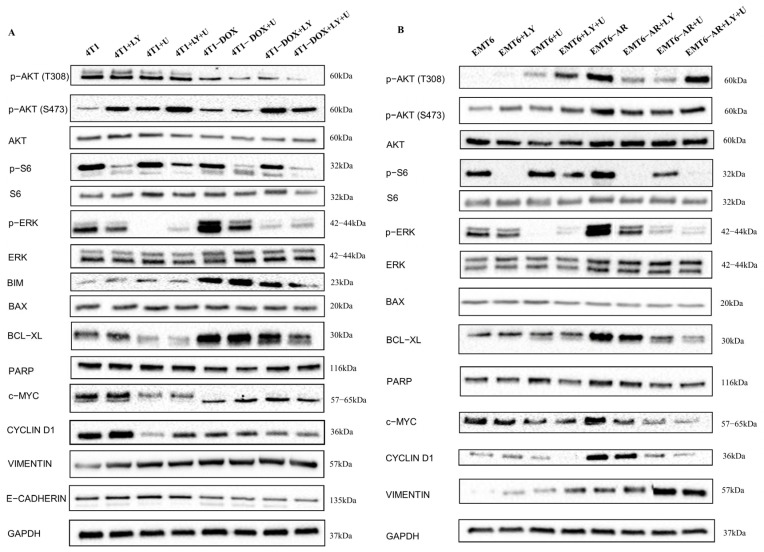
Changes in expression levels of proliferation-, survival-, cell-cycle-, apoptosis- and EMT-related proteins in DOX-sensitive and -resistant 4T1 (**A**) and EMT6 (**B**) cells after treatment with PI3K and MEK inhibitors at 24 h as determined by Western blot analysis.

We examined the expression of multiple markers using Western blot analysis to investigate intracellular signaling changes at the protein level (Figure 4) at 24 h. In DOX-resistant 4T1 cells, all treatments led to a decrease in p-AKT (T308) compared to parental cells. Additionally, p-AKT (S473) levels were significantly reduced in the DOX-resistant 4T1 cells, except for MEK inhibitor treatment. On the other hand, the expression profile of p-AKT(T308) and p-AKT(S473) in EMT6 and EMT6-AR cells was the opposite of observed in the 4T1 group. Although LY294002 and U0126 treatments led to a decrease in the expression of these markers, they were upregulated in the DOX-resistant EMT6 cells compared to parental EMT6 cells. p-S6, a readout of the PI3K/AKT signaling pathway, was decreased in both LY294002-treated parental 4T1 and EMT6 cells, as well as their corresponding DOX-resistant groups. Similarly, p-MEK, a marker for MAPK pathway inhibition, was downregulated in all U0126-treated parental or resistant cell groups. Nevertheless, our attention was drawn to the significant upregulation of p-ERK in both 4T1 and EMT6 DOX-resistant control cells compared to their parental counterparts, which may explain the increased aggressiveness of the DOX-resistant cells.

Regarding apoptotic pathways, we observed elevated BIM, BAX, BCL-XL and PARP expressions in all cells. In 4T1 cells, BIM and BCL-XL were upregulated in DOX-resistant groups, while BAX and PARP expressions were downregulated in the same groups. In EMT6 cells, BIM expression was undetectable; however, BCL-XL was upregulated, and BAX was downregulated in EMT6-AR cells, which may explain their enhanced survival behavior.

For the cell cycle markers, we assessed c-MYC and CCND1 expressions. In 4T1 cells, both c-MYC and CCND1 were less expressed in DOX-resistant groups. Conversely, in EMT cells we observed the opposite trend, with both cell cycle markers being highly expressed in EMT6-AR cells. Nonetheless, it should be noted that the inhibitor treatments, particularly when combined, had a more pronounced effect on c-MYC and CCND1 expressions in the DOX-resistant groups.

At the outset of this study, we observed both morphological and molecular changes associated with EMT in the DOX-resistant cells. Subsequently, it became essential to assess the expression of EMT markers through the application of pathway inhibitors. We observed significant and consistent changes in Vimentin expression in 4T1 cells, as E-Cadherin expression decreased with DOX resistance. In EMT6 cells, E-Cadherin expression was undetectable; however, Vimentin expression significantly increased in EMT6-AR cells. Notably, the expression was further elevated in the DOX-resistant group treated with U0126 and combined U0126 and LY294002, suggesting that this treatment could potentially induce EMT.

**Figure 5:**
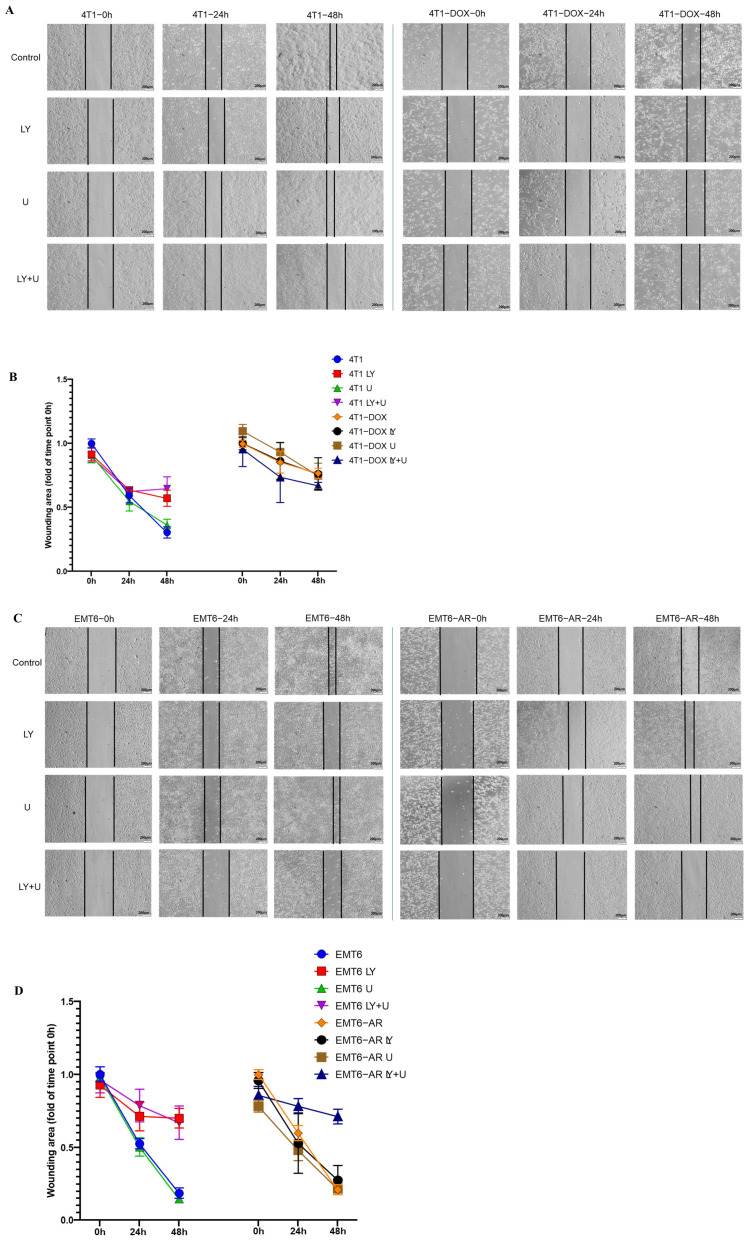
Wound healing assay demonstrating the effects of PI3K and MEK inhibitors on the migration of DOX-sensitive and -resistant 4T1 (**A**) and EMT6 cells (**C**). Scratch closure was assessed at 0, 24, and 48 h post treatment in 4T1 and 4T1-DOX cells (**B**) and in EMT6 and EMT6-AR cells (**D**). Data were normalized to the respective control within each group.

Following these compelling results, we conducted a scratch assay to measure migration capacity in all treatment groups for 24 and 48 h (Figure 5). LY294002 and combined LY294002 and U0126 treatments resulted in reduced migration compared to the parental cells for 4T1 cells. However, in DOX-resistant 4T1 cells, similar closure rates were observed between the treatment groups and their respective control groups (Figure 5A,B). For the EMT6 parental cells, Western blot data revealed that the treatments, particularly combined LY294002 and U0126, increased Vimentin expression. However, upon examining migration data, we observed a reduction in migration with LY294002 and combined LY294002 and U0126 treatments. In EMT6-AR cells, although Western blot data showed a significant increase in Vimentin expression with U0126 and combined LY294002 and U0126 treatments, the wound closure was more prominent in the combined-LY294002-and-U0126-treated cells compared to their respective control group, where the wound remained more open (Figure 5C,D).

## 3. Discussion

Among breast cancer subtypes, TNBC accounts for roughly 15% of all and is associated with the worst prognosis. Despite efforts, progression-free survival and overall survival have not been significantly improved [28]. Drug resistance in TNBC is caused by a variety of molecular processes. Identification of these mechanisms is crucial since it can both improve treatment and prognosis for TNBC patients. DOX, a DNA-intercalating anthracycline, remains a primary treatment option for early-stage breast, ovarian, lymphoma, and leukemia cancers. It is also currently the most widely used and effective chemotherapy drug for treating breast cancer [6]. However, it has been demonstrated that it can lead to drug resistance and even promote tumor growth, recurrence, and metastasis, ultimately contributing to poor prognosis and survival outcomes for patients [29,30]. Despite extensive research into various mechanisms, DOX resistance continues to be a significant and unresolved challenge in cancer treatment.

The causes of DOX resistance may stem from a variety of mechanisms. At the root of these mechanisms, genomic and epigenomic differences, as well as varying durations and intensities of exposure to environmental factors, could play a significant role. These underlying differences can influence a wide range of components, including drug efflux, cellular signaling pathways, DNA repair mechanisms, autophagy, apoptotic pathways, and the immune system [31,32].

Ate et al., in an intriguing novel study on DOX resistance, investigated the molecular mechanisms underlying ZC3H13-mediated ferroptosis in TNBC. They observed the downregulation of ZC3H13 in DOX-resistant cells. The overexpression of ZC3H13 decreased resistance, suppressed proliferation, and induced ferroptosis, which in turn inhibited KCNQ1OT1/TRABD, thereby restraining the growth of DOX-treated tumors in vivo [33]. This study demonstrated that not only apoptosis, necrosis, or autophagy but also many other cell death pathways may be involved in resistance. In our study, we aimed to examine different cell death pathways and the interactions between these pathways, as well as their impact on drug resistance in TNBC cell lines.

Smoots et al. explored bocodepsin (OKI-179), a class I histone deacetylase (HDAC) inhibitor, in overcoming DOX resistance in TNBC. They examined the combination of bocodepsin and DOX, showing that bocodepsin enhances doxorubicin’s effectiveness by promoting apoptosis and decreased senescence. This combination led to synergistic antiproliferative effects in vitro and improved tumor inhibition in vivo. Additionally, this combined treatment proved to be effective regardless of p53 mutation status, highlighting its potential to address resistance in TNBC patients [34]. However, the fundamental difference in their study is that, unlike ours, they did not expose the human TNBC cell lines to prolonged DOX treatment. Instead, they selected the cells with the highest survival rates after 72 h of exposure.

On the other hand, Chen et al. used long-term DOX-resistant MDA-MB-231/ADR cells and human tissues and identified TXNIP (thioredoxin-interacting protein), a protein involved in the regulation of cellular redox balance and oxidative stress. It was found that TXNIP expression is significantly downregulated in MDA-MB-231/ADR cells and reintroducing TXNIP into these cells promotes an increase in reactive oxygen species (ROS) levels. Elevated ROS levels subsequently cause DNA damage in these cells, which disrupts their survival mechanisms and sensitizes them to DOX treatment [35]. Although we did not assess TXNIP levels in the resistant cells that we used in our study, it is plausible that the downregulation of TXNIP could be associated with enhanced activation of AKT or MEK, thereby promoting increased cell proliferation and survival. This could potentially serve as a mechanism underlying chemotherapy resistance in our study.

Also, Paramanantham et al. demonstrated that DOX-resistant MDA-MB-231 cells acquired cancer-stem-cell-like properties and EMT features and revealed that the resistant cells secrete signaling molecules that trigger autocrine signaling, enabling the transfer of the resistant phenotype to parental cells. This autocrine signaling cascade facilitates the acquisition of cancer-stem-cell-like traits and EMT characteristics in the originally sensitive TNBC cells, leading to the spread of resistance within the tumor population [36]. Their study is consistent with ours, as resistant cells demonstrated upregulation of Cyclin D1, MMP2, and MMP9 and downregulation of E-CADHERIN. Similarly, they observed upregulation of AKT and ERK1/2 in resistant cells due to EGFR activation and proposed the critical role of autocrine signaling in the propagation of drug resistance.

In DOX-resistant MDA-MB-231 cells, Wei et al. highlighted the role of cFLIP in mediating doxorubicin resistance and identified HuR as a positive regulator of cFLIP. They showed that the overexpression of cFLIP reduced the activation of Caspase-8 and the cleavage of PARP in DOX-sensitive cells. In contrast, apoptosis was induced by siRNA-mediated cFLIP knockdown, with resistant cells exhibiting a more apoptotic phenotype. They suggested that DOX resistance in breast cancer cells is largely caused by cFLIP overexpression and its regulator HuR, underscoring the possibility of blocking cFLIP expression and inhibiting HuR as a potential therapeutic target to overcome cFLIP-mediated chemoresistance [37]. Consistent with their study, we observed overexpression of BCL-XL in both DOX-resistant 4T1 and EMT6 cells, suggesting that the activation of anti-apoptotic proteins could serve as potential targets for overcoming resistance.

In our study, we demonstrated that DOX resistance can enhance various survival and signaling pathways. When we inhibited MEK in the signaling pathways activated by DOX resistance, for instance, in migration assays, the application of an MEK inhibitor led to an enhanced cell migration. Similarly, in proliferation assays, the treated cells either proliferated to the same extent as the control group or even exceeded it. This aggressive phenotype may be due to the activation of alternative signaling pathways, such as PI3K/AKT [38] or other pathways that were not explored in our study [39], and was also observed through the significant upregulation of MMP2 and MMP9 expression in the resistant cells, as analyzed by RNA expression. Also, the reactivation of several RTKs upstream of the MAPK pathway, which initiates the signaling cascade that ultimately results in cellular growth and proliferation and resistance, is another significant consequence of MEK inhibition [40]. Therefore, by using a PI3K inhibitor to block another significant survival pathway that could be activated, we obtained more substantial results in proliferation, migration, gene and protein expression, when both inhibitors were applied together in the context of DOX resistance. Continuing in this context, loss of PTEN or PIK3CA mutations has been associated with intrinsic resistance to MEK inhibitors in various cancers [41,42]. In addition, RTKs like EGFR, ERBB3, IGF-1R, or PDGFR can be quickly reactivated in response to MEK inhibition [43,44]. Efforts to diminish these activated signaling pathways may result in more profound alterations in cancer progression. It should always be considered that cancer’s inherent instability and plasticity may facilitate the adaptation to various detrimental attempts, thereby revealing more aggressive phenotypes in some cases [45].

To further investigate the causal relationship between mitophagy and DOX resistance in murine TNBC, we analyzed the expressions of PINK1 and PARKIN. However, we did not observe consistent changes in their expression across different treatment groups or cell types. Similarly, Yan et al. reported a rapid increase in BNIP3L protein levels in DOX-treated HCT8 CSCs, while PINK1 and PARKIN expression remained unchanged [46].

One significant limitation of this study is the lack of in vivo validation, which could offer a more physiologically relevant context and provide additional support for our hypothesis. Although complementary approaches such as genetic silencing (e.g., siRNA-mediated knockdown), functional invasion assays to further substantiate EMT, and direct assessment of matrix MMP activity would provide additional mechanistic and functional validation, our work was primarily designed to evaluate molecular and signaling alterations associated with pathway modulation using a pharmacological approach. Nevertheless, we acknowledge that incorporating genetic loss-of-function strategies, invasion assays, and MMP activity measurements would further strengthen the mechanistic depth of the findings, and we propose these as important directions for future investigations.

## 4. Materials and Methods

### 4.1. Gene Expression Data Acquisition and Processing

The GSE222984 gene expression dataset was obtained from the Gene Expression Omnibus (GEO) database (https://www.ncbi.nlm.nih.gov/geo/, accessed on 11 April 2025) maintained by the National Center for Biotechnology Information (NCBI), which is a publicly available database. The gene expression data was analyzed using the GEO2R tool. Samples were grouped according to DOX-resistant TNBC MDA-MB-231 cells and parental MDA-MB-231 cells. GSE202536 was also obtained from the Gene Expression Omnibus (GEO) database. Samples were grouped as CAL51-naive cells vs. CAL51DOX (resistant to 0.4 µM DOX). Differentially expressed genes (DEGs) were identified using GEO2R and the top 250 genes were selected based on adjusted *p*-value < 0.05. These genes were uploaded to the Enrichr online enrichment analysis tool (https://maayanlab.cloud/Enrichr/, accessed on 11 April 2025) to identify significantly enriched pathways. The MSigDB (Molecular Signatures Database) gene set library was used for the enrichment analysis. Pathways were ranked by adjusted *p*-value and combined score, and the top enriched pathways were selected for interpretation.

### 4.2. Survival Analysis

The prognostic value of Vimentin, MMP9 and CDH2 expressions was evaluated using the Kaplan–Meier Plotter (KMplot; https://kmplot.com/analysis/, accessed on 11 April 2025) database. The analysis was performed in the TNBC cohort. Kaplan–Meier survival curves were generated to assess the association between Vimentin, MMP9, and CDH2 expression and overall survival. In addition, 95% confidence intervals (CIs) and log-rank *p*-values were calculated automatically by the KMplot tool.

### 4.3. Cell Culture

4T1, EMT6 and EMT6-AR TNBC murine breast adenocarcinoma cell lines were kindly provided by the Esendagli Lab (Hacettepe University). 4T1-DOX resistance cells were generated via continuous treatment with increasing concentrations of DOX, doubled every two weeks, beginning at 25 nM and progressively increased up to a final concentration of 6400 nM, at which point the escalation was terminated. EMT-6 cells were kept in 1 µM DOX-containing media. 4T1, 4T1-DOX, EMT6 and EMT6-AR cells were cultured in DMEM (Gibco, Waltham, MA, USA) supplemented with fetal bovine serum (10%) (Gibco, Waltham, MA, USA), 100 U/mL penicillin, 100 µg/mL streptomycin and non-essential amino acids (0.1 mM) (Sigma Aldrich, Saint Louis, MO, USA). To ensure cell adherence and adequate gas exchange, the cells were cultivated in sterile flasks at 37 °C in a humidified incubator with a 5% carbon dioxide environment. The cells were trypsinized using a trypsin/EDTA solution (Sigma Aldrich, Saint Louis, MO, USA) once they reached 70% confluence. To maintain culture continuity, the cells were then diluted in fresh media at a ratio of 1:4. Doxorubicin was obtained from SABA in a 50 mg lyophilized stock form and was dissolved in dH_2_O to achieve a stock concentration of 100 µM.

### 4.4. Cell Viability Assays

Cell viability was determined using a colorimetric MTT (3-(4,5-Dimethylthiazol-2-yl)-2,5-Diphenyltetrazolium Bromide) (Merck cat: 475989) assay. Cells were seeded in 96-well plates at 2000 cells/mL and on the next day treated with or without 10 μM PI3K inhibitor (LY294002, CST, cat. no: 9901P) or 10 μM MEK inhibitor (U0126, Cayman, cat. no: 70970). After 24, 48 and 72 h, a MTT assay was performed according to the manufacturer’s instructions. Color absorbance was measured using a microplate reader (SpectraMax iD3) at a wavelength of 570 nm. Results were normalized against the mean measurements from at least six replicates in the control group. Each experiment was repeated twice.

### 4.5. RNA Isolation, cDNA Synthesis and RT-qPCR

Total RNA was isolated with a GENEALL RNA isolation kit (Catalog number: 305-101) according to the manufacturer’s protocol and quantified with a Nanodrop. RNAs were transcribed into cDNA by using a Roche Transcriptor High Fidelity cDNA (Complementary DNA) Synthesis Kit (Catalog number: 5091284001). Following cDNA synthesis, RT-qPCR (Real-Time Polymerase Chain Reaction) was done using SYBR green (Biorad) with a Biorad CFX Connect Real-Time System. All gene expressions were normalized to Beta Actin as a reference gene. Expression levels of the indicated genes were calculated by the ΔΔCt method and graphs were plotted by using log2 fold changes for each gene, which were normalized against the control group. The primers used in this study are listed in Supplementary Table S1.

### 4.6. Western Blot

At the end of the incubation time for each indicated inhibitor concentration, cell pellets were obtained from the cells, which were seeded 2 × 10^5^ per 6-well plate. Using these pellets and the newly made RIPA buffer (NaCl, TrisHCl, NP-40, 10% SDS), protease inhibitor cocktail (CST, 5871S), and phosphatase inhibitor cocktail (CST, 5870S), proteins were extracted. The BCA method was used to evaluate the total protein concentration using a protein assay reagent kit (Thermo Scientific, Waltham, MA, USA, 23227). Proteins were denatured using 4X Loading Dye (Biorad, Hercules, CA, USA, 161-0747) for five minutes at 95 °C prior to loading onto the gel. Then, 30 µg of protein from each sample was placed onto a 10% SDS-PAGE (Bio-Rad Acrylamide Kit, 161–0183) and electrophoretically separated in the running buffer. Proteins were transferred onto a PVDF membrane (Merck, 3010040001) using the semi-dry transfer method. Membranes were then blocked for one hour at room temperature using 5% milk made with Tris-buffered saline and 0.2% Tween-20 (TBST). After that, membranes were incubated with the antibodies for one night at 4 °C at a dilution of 1:1000, unless otherwise specified. Following three TBST washes for ten minutes, the membranes were incubated with secondary antibodies (1:5000) (anti-rabbit CST #7074) for one hour at room temperature. After that, they were washed three times for 10 min in TBST. The membranes were developed in ECL (BioRad Clarity ECL Substrate, Hercules, CA, USA #17050060) and images were taken with Chemidoc (BioRad, Hercules, CA, USA). The primary antibodies used in this study are as follows: p-AKT (T308) CST #13038, p-AKT (S473) #4060, AKT CST #4691, p-S6 CST #4885, S6 CST #2217, p-ERK CST #4370, ERK CST #4695, BIM CST #2819, BAX CST #2772, BCL-XL CST #2762, PARP CST #9532, c-MYC CST #5605, CYCLIND1 CST #2922, VIMENTIN CST #5741, E-CADHERIN CST #3195, and GAPDH CST #2118.

### 4.7. Wound Healing Assay

All cells were seeded (2 × 10^5^ cells per 6-well plate) to evaluate their migratory capacity. The following day, vertical wounds were inserted using a 200 μL sterile pipette tip, and an Olympus CKX53 was used to take pictures of them. Additionally, photos were obtained after 24 and 48 h. At least six pictures were taken each time. Olympus cellSens Entry software 1.16 was used to measure the distance between the two sides of the wound.

### 4.8. Statistical Analysis

Statistical analysis was performed using GraphPad Prism 9 (GraphPad Software, San Diego, CA, USA) with the use of appropriate tests for each experiment. *p*  <  0.05 was considered statistically significant.

## 5. Conclusions

This study explored the effects of inhibiting activated signaling pathways in DOX-resistant TNBC cells, with a particular focus on their influence on MAPK and PI3K/Akt signaling cascades, cell cycle regulation, and key regulators involved in the epithelial–mesenchymal transition (EMT) process.

Although targeting signaling pathways involved in DOX resistance may yield more aggressive outcomes, depending on the context, it is important to identify the significant factor or molecule to overcome resistance. Future research is anticipated to validate the biomarkers that were found to be able to predict resistance to chemotherapeutic drugs. Molecular attribution and detailed testing of the phenotypic switch of cancer cells under treatment are needed to develop more personalized and efficient treatments for TNBC patients. In both early and advanced disease states, the therapeutic approach may be guided by these prognostic indicators.

## Data Availability

The original contributions presented in this study are included in the article/Supplementary Materials. Further inquiries can be directed to the corresponding author.

## Supplementary Materials

The following supporting information can be downloaded at https://www.mdpi.com/article/10.3390/ijms27062792/s1.
